# Wearables for gait and balance assessment in the neurological ward - study design and first results of a prospective cross-sectional feasibility study with 384 inpatients

**DOI:** 10.1186/s12883-018-1111-7

**Published:** 2018-08-16

**Authors:** Felix P. Bernhard, Jennifer Sartor, Kristina Bettecken, Markus A. Hobert, Carina Arnold, Yvonne G. Weber, Sven Poli, Nils G. Margraf, Christian Schlenstedt, Clint Hansen, Walter Maetzler

**Affiliations:** 1 Department of Neurology and Neurodegenerative Diseases and Hertie Institute for Clinical Brain Research, University Tübingen, 72076 Tübingen, Germany; 2 DZNE, German Center for Neurodegenerative Diseases, Tuebingen, Germany; 3 Department of Neurology, University Hospital Schleswig-Holstein, Campus Kiel, Arnold-Heller-Str. 3, Haus 41, 24105 Kiel, Germany; 4 Department of Neurology and Epileptology, University Tübingen, 72076 Tübingen, Germany; 5 Department of Neurology & Stroke, University Hospital Tübingen, Tübingen, Germany

**Keywords:** Accelerometer, Inertial sensor, Postural control, Neurological diseases

## Abstract

**Background:**

Deficits in gait and balance are common among neurological inpatients. Currently, assessment of these patients is mainly subjective. New assessment options using wearables may provide complementary and more objective information.

**Methods:**

In this prospective cross-sectional feasibility study performed over a four-month period, all patients referred to a normal neurology ward of a university hospital and aged between 40 and 89 years were asked to participate. Gait and balance deficits were assessed with wearables at the ankles and the lower back. Frailty, sarcopenia, Parkinsonism, depression, quality of life, fall history, fear of falling, physical activity, and cognition were evaluated with questionnaires and surveys.

**Results:**

Eighty-two percent (*n* = 384) of all eligible patients participated. Of those, 39% (*n* = 151) had no gait and balance deficit, 21% (*n* = 79) had gait deficits, 11% (*n* = 44) had balance deficits and 29% (*n* = 110) had gait and balance deficits. Parkinson’s disease, stroke, epilepsy, pain syndromes, and multiple sclerosis were the most common diseases. The assessment was well accepted.

**Conclusions:**

Our study suggests that the use of wearables for the assessment of gait and balance features in a clinical setting is feasible. Moreover, preliminary results confirm previous epidemiological data about gait and balance deficits among neurological inpatients. Evaluation of neurological inpatients with novel wearable technology opens new opportunities for the assessment of predictive, progression and treatment response markers.

## Background

Gait and balance deficits occur in many neurological diseases. The evaluation of these deficits at the wards of hospitals is often based on qualitative parameters collected by the treating physicians and allied health professionals or on semi-quantitative scoring tools. For example, in Parkinson’s disease (PD), the Unified Parkinson Disease Rating Scale (MDS-UPDRS) is regularly used to rate motor symptoms including gait and postural stability [1]. While such scales, questionnaires and surveys have been subject to multiple validation studies, they have limitations regarding inter-rater variability and subjectivity [2–5].

With the recent and ongoing development of wearables (mainly in the sport and fitness sectors), this technology has reached a sophisticated level making it interesting for medical purposes [6–16]. A particularly relevant field is the complementary assessment of inpatients at neurological wards, as wearables are specifically capable of assessing gait and balance deficits which are common in neurological patients [15, 17].

Only a small number of studies have investigated feasibility and acceptability of wearables in an inpatient setting, with limitations such as small sample sizes [18] and the investigation of only one disease [19]. This study aims to investigate the feasibility and usefulness of wearables during clinical evaluation in a large sample of neurological inpatients.

## Methods

### Participants

All inpatients referred to the three normal wards of the Neurology Center at the University Hospital of Tübingen between 09/2014 and 04/2015 (16-week assessment periods for each ward) were asked to take part in the study if they were between 40 to 89 years of age (this selection criterion was chosen due to feasibility issues) and were able to walk with or without walking aid. Exclusion criteria were the inability to give informed consent, a fall frequency of more than one fall per week (risk of falls during the assessment too high), and impaired cognition as defined by a Mini Mental State Examination (MMSE) score below 10 points. Participants who had at least one fall during the last 2 years were defined as fallers. The ethics committee of the medical faculty of the University of Tübingen approved the study (No. 356/2014BO2) and all participants gave written informed consent prior to participation.

### Quantitative gait and balance assessment

Participants were equipped with a wearable sensor system (Rehawatch®, Hasomed, Magdeburg, Germany) consisting of three sensor-units worn at both ankles and at the lower back (L4-L5) [20]. Each sensor-unit contains 3D accelerometers (±8 g), 3D gyroscopes (±2000°/s) and 3D magnetometers (±1.3Gs) resulting in nine degrees of freedom and the raw data was processed and analyzed using validated and company provided algorithms [21]. The assessment included the following tasks: Participants walked seven times a 20 m distance under single (slow, comfortable, and fast speed) and dual tasking conditions (checking boxes and subtracting serial 7 s during comfortable and fast walking) [22, 23]. Static balance during quiet standing at the center of stability was tested on flat ground with four different positions of the feet for 30 s each: open stance with feet placed in parallel position with 5–10 cm in between, closed stance (parallel position), semi-tandem stance, and tandem stance [24]. The task one difficulty level below the one successfully performed on flat ground was then performed for 30 s on a foam pad (Airex balance pad, 50x41x6 cm). Static balance at the limit of stability was tested with an adapted version of the Functional Reach Test [25] over a 15 s period. Overall mobility and transfer was tested with the Timed-Up-and-Go test (TUG) under comfortable and fast speed conditions [26–28]. Muscle strength was assessed with a hydraulic hand dynamometer (DanMic Global®, San Jose, USA) and muscle mass with bioimpedance (Akern Bia 101, SMT medical GmbH&Co. KG, Würzburg, Germany).

### Assessments with scales and questionnaires

Fear of falling was assessed with the German version of the Falls Efficacy Scale-International (FES-I) [29]. Self-concepts of health, activity, cognition, social support and risk factors for age-associated diseases were assessed [30]. Depression was evaluated with the German version of the Beck’s Depression Inventory II (BDI-II) [31, 32]. Health-related quality of life was assessed with the EQ-5D-5 L. This scale addresses mobility, autonomy, pain, fear, despondence, daily living activities and health [33]. The MMSE [34] and the Trail Making Test (TMT) [35] were used to assess cognition, and part III of the Movement Disorders Society-sponsored Unified Parkinson’s Rating Scale (MDS-UPDRS) [1] was used for the assessment of motor symptoms. Function of the sensory nerves was assessed at the medial malleoli of the lower extremities and the basal joint of each thumb with a Rydel Seiffer tuning fork.

### Classification of impaired gait and balance

A gait deficit was defined as > 15% lower walking speed compared to mean age-corrected speed according to [36, 37]. Presence of a balance deficit was considered when tandem stance could be performed no longer than 10 s [38, 39].

### Statistical analysis

Statistical analysis was conducted with JMP 11.1.1 (SAS). Demographic data of the different groups were compared with Kruskal-Wallis-test (or Fisher’s exact test for categorical data). Post hoc testing was performed with Mann-Whitney-U test. *P* values below 0.05 were considered significant. Bonferroni correction for multiple testing was applied for post-hoc tests (*p* < 0.0083).

## Results

**Fig. 1:**
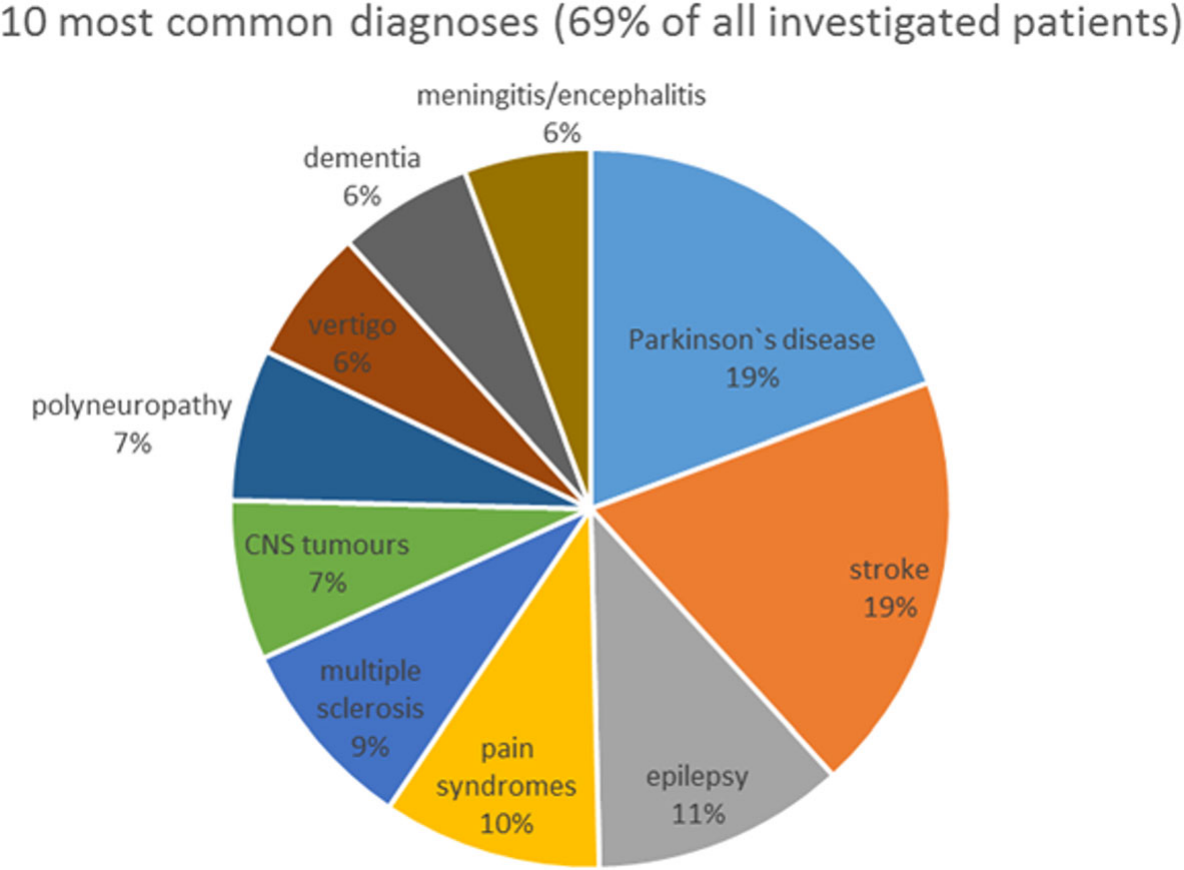
Graphical representation of the ten most common diagnoses within the 384 study participants

Of 468 inpatients eligible for the study (i.e., fulfilled all inclusion criteria and no exclusion criterion, and were not excluded due to logistic reasons), 384 (82%) participated. Of those, 60% were male. Mean age of the cohort was 62 years. The 10 most common diagnoses (69% of all investigated patients) were Parkinson’s disease (PD, *n* = 51), stroke (*n* = 50), epilepsy (*n* = 30), pain syndromes (*n* = 26), multiple sclerosis (MS, *n* = 23), CNS tumours (*n* = 19), polyneuropathy (*n* = 18), vertigo (*n* = 16), dementia (n = 16), and meningitis/encephalitis (*n* = 15). During the study, no severe adverse events occurred (Fig. 1).

One hundred and 51 participants (39%) had no gait and balance deficit (control group), 79 (21%) had a gait deficit, 44 (11%) had a balance deficit and 110 (29%) had a gait and balance deficit. The highest proportion of patients with gait deficits (33%) was found in the meningitis/encephalitis cohort, the highest proportion of patients with balance deficits (17%) in the MS cohort, and the highest proportion of participants with gait and balance deficits (41%) in the PD cohort. Patients with pain syndromes had rarely gait and/or balance deficits.

**Fig. 2:**
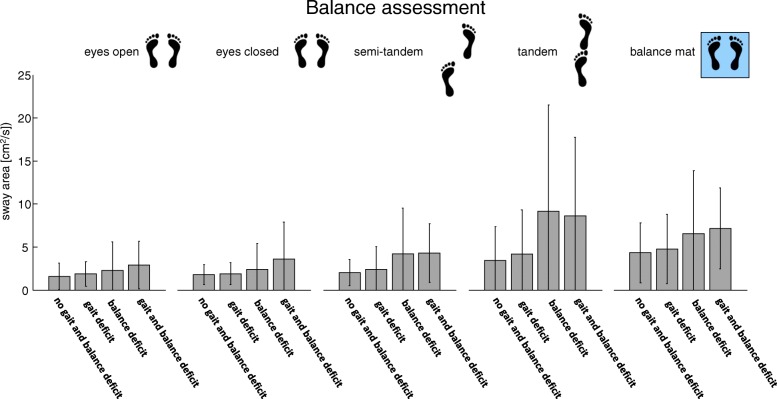
During the balance assessment the participants with a balance or gait and balance deficit show the largest sway area compared to the controls and the patients with the gait deficit

**Fig. 3:**
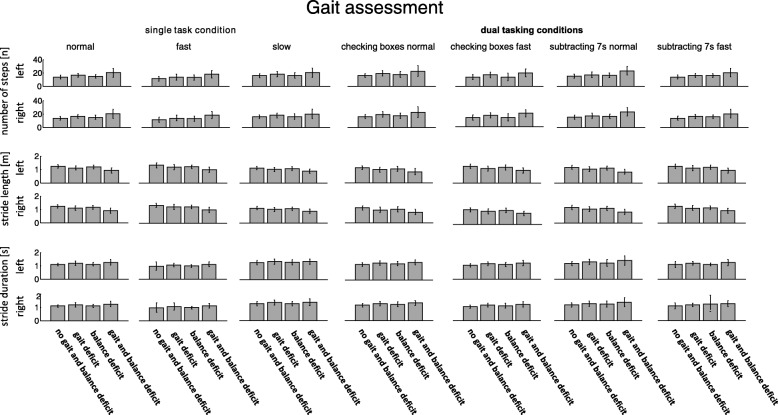
During the gait assessments (single and dual task conditions) the participants with a gait or gait and balance deficit show the largest number of steps, the smallest stride length and the highest stride duration compared to the controls and the participants with a balance deficit

**Fig. 4:**
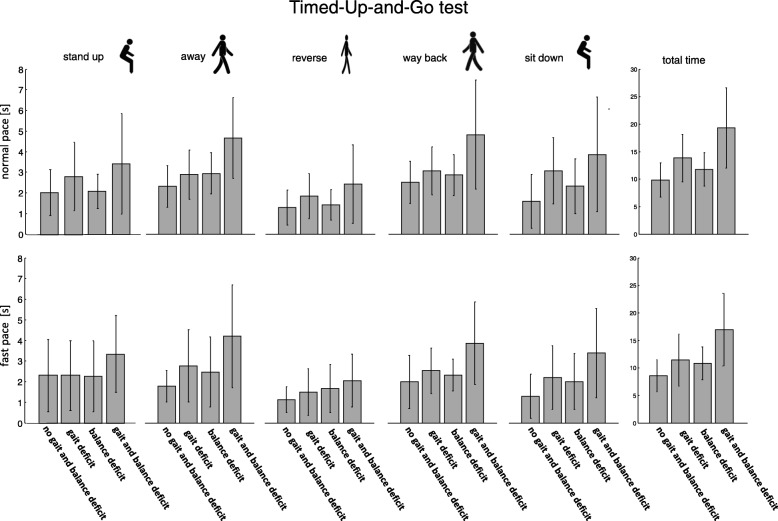
The TUG tests shows a similar pattern and the duration of the individual phases is increased for participants with a gait or gait and balance deficit compared to the controls and the participants with a balance deficit

Patients complied well with the quantitative gait and balance assessment and descriptive results from the balance, gait and postural transitions are shown in Figs. 2, 3 and 4.

MMSE and TMT performances were significantly worse in all cohorts with gait and/or balance deficits, compared to the control cohort. Moreover, the cohort with gait and balance deficits performed worse in the TMT compared to the group with gait deficits. TUG durations were slower in the cohort with gait and balance deficits than in both the gait deficit cohort and the balance deficit cohort, and fastest in the control cohort. The same pattern was observed with regard to fear of falling, with highest FES-I values (i.e. the highest fear of falling) in the gait and balance deficits cohort. BDI II values were higher in the cohort with gait and balance deficits, compared to the control cohort. The cohort with gait and balance deficits had lower grip force when compared to the cohort with gait deficits and the control cohort. A more detailed description of the inter-cohort comparisons is presented in Table 1.

**Table 1:** Demographic, clinical, and semiquantitative/quantitative study outcomes of the whole cohort, as well as of the subcohorts with and without gait and balance deficits

	Whole cohort (*N* = 384)		Controls (*N* = 151)		Gait deficits (*N* = 79)		Balance deficits (*N* = 44)		Gait and balance deficits (*N* = 110)		*P*-Value
	Median	Range	Median	Range	Median	Range	Median	Range	Median	Range	
Age [years]	64	40–90	57	40–86	60	40–89	70*^#^	44–89	69.5*^#^	41–90	< 0.0001
Gender [% female]	42.4		43.0		36.7		43.2		45.5		0.67
Height [m]	1.72	1.48–2.01	1.73	1.49–2.01	1.73	1.48–1.98	1.70	1.58–1.88	1.70	1.49–2.00	0.04
Weight [kg]	79	37–134	80	50–134	82	46–117	79	55–115	74	37–123	0.13
BMI [kg/m^2^]	26.2	14.9–43.0	26.3	19.4–41.8	26.4	17.3–41.6	26.8	19.4–38.4	25.9	14.9–43.0	0.77
Falls in the last 24 months [N]	0	0–100	0	0–50	0	0–50	1*	0–55	1*^#^	0–100	< 0.0001
At least one fall in the last 24 months [%]	46		29		42		62*		65*^#^		< 0.0001
LACHS (0–15)	3	0–10	2	0–8	3*	0–6	3*	1–9	4*^#^	0–10	< 0.0001
MMSE (0–30)	28	13–30	29	24–30	28*	13–30	28*	13–30	27*	13–30	< 0.0001
TMT-A [s]	49	13–300	38	13–300	48*	23–300	55*	26–300	72*^#^	17.7–300	< 0.0001
TMT-B [s]	149	34–300	101	34–300	129*	38–300	174*	60–300	300*^#^	38.8–300	< 0.0001
∆TMT [s]	85	−30-280	60	−30-280	78*	0–253	98	0–257	149*	0–270	< 0.0001
Timed up and go convenient speed [s]	12	6–92	10	6–25	12*	8–28	11*	8–18	16*^#+^	8–92	< 0.0001
Timed up and go fast speed [s]	9	5–47	7	5–15	10*	6–22	11*	6–15	14*^#+^	7–47	< 0.0001
BDI II (0–63)	10	0–51	8	0–51	10	0–28	10	0–38	12*	0–51	0.0004
FES-I (0–64)	20	0–64	18	0–63	21*	0–44	20*	14–48	27*^#+^	14–64	< 0.0001
EQ5D VAS (0–100)	60	1–100	70	20–100	55	10–95	50*	5–90	50*^#^	1–95	< 0.0001
Functional Reach [cm]	23	3–82	27	8–45	23*	3–82	20*	5–35	18*^#^	5–34	< 0.0001
Gait speed [m/s]	1.10	0.27–2.33	1.34	0.95–2.33	0.99*	0.56–1.67	1.15*^#^	0.81–2.03	0.80*^#+^	0.27–1.5	< 0.0001
Grip force [kg]	27	3–76	29	10–76	29	7–56	28	15–51	23*^#^	3–51	< 0.0001

Data is presented with median and range. *P*-values were calculated using the Kruskal-Wallis-test, with post hoc Mann-Whitney-U-Test and Chi^2^ test. For post hoc testing Bonferroni correction for multiple testing was applied. * *p* < 0.0083 for comparison with the control cohort group, ^#^*p* < 0.0083 for comparison with the gait deficit cohort, ^+^*p* < 0.0083 for comparison with the balance deficit cohort. *BDI II* Beck’s depression inventory II, *BMI* Body mass index, *EQ5D VAS* Visual analog scale of the EuroQol-5 dimension questionnaire, *FES-I* Falls efficacy scale international, *LACHS* Geriatric screening according to Lachs et al., *MMSE* Mini-mental state examination, *TMT* Trail making test (part A, B, and B-A = ∆TMT)

## Discussion

In the presented study, we performed routine clinical gait and balance assessments complemented by an exhaustive evaluation of geriatric parameters in a neurological department at a university hospital. To the best of our knowledge, this is the first sensor-based cross-sectional study in a clinical environment of a university hospital, covering a wide range of neurological diseases. Our overall cohort represents a wide range and representative number of neurological diseases. A study with a similar setting but without sensor-unit-based assessments displayed a comparable composition of neurological diseases [17] with the five most common diagnoses completely overlapping.

Acceptance of sensor-unit-based assessments in our study was high. Only 18% of eligible patients refused to take part in the study. We did not experience any logistical issues during the assessments. The sensor system was easy and quick to apply and none of the patients felt restricted by the sensor system. Moreover, no serious adverse events, e.g. falls, occurred. These results bode well for the clinical uptake of wearable sensors into regular care.

Not surprisingly, frequencies of gait and balance deficits of neurological inpatients are higher compared to observations in the community and in outpatient clinics. However, these deficits are ubiquitously observed. In a community-based study investigating 467 participants, the prevalence of gait deficits was 14% in those between 67 and 74 years of age, 29% in those between 75 and 84 years and 49% in those 85 years and older [40]. In a cross-sectional investigation of 488 community residing adults aged between 60 and 97 years, 32% of the cohort presented with impaired gait and the prevalence increased with age. However, 38% of the subjects aged 80 years and older still had a normally preserved gait [41]. In outpatients clinics, gait deficits occur in 35% of patients, most of them having neurological causes [42].

It is of note that, in our study, the cohort with gait (but not balance) deficits was of similar age as the control cohort. This suggests that the slower gait speed of this group was not induced by an overall decline in performance due to aging, but rather due to the underlying disease processes. This could be an interesting observation in the light of ongoing studies investigating gait speed as a relevant outcome parameter for disease and disability. Moreover, groups with gait and/or balance deficits showed impaired cognitive performance compared to the control group, supporting the association between motor performance and cognition [43–45]. It is also of note that not only the balance deficit cohort but also the gait deficit cohort performed worse than the controls in the functional reach test. This finding supports the link between static balance and gait and reflects the various aspects of postural control which should be further investigated in future studies.

The current study has several limitations. First, only gait velocity was used to define gait deficits. Although reduced gait speed impacts patients’ mobility, there are several additional gait variables (e.g. gait variability, asymmetry) which are important features as they are associated with fall risk [46–49]. However, numerous more sophisticated algorithms are currently developed and validated allowing investigation of the multidimensional aspects of postural control (e.g., [20, 50, 51]) in more detail. Including dynamic, proactive, and reactive postural control parameters will give a broader view of the multidimensional aspects of balance control and help understand different pathologies of the diseases. This aspect is currently the focus of a more detailed sensor-unit-based analysis of the dataset.

## Conclusion

In conclusion, this study shows that the use of inertial sensors in a clinical setting by investigating patients in neurological wards of a university hospital over a time of 16 weeks is feasible. These results should motivate to further design inpatient assessments using wearable technology, and of collaborative projects using such datasets for further in-depth analyses.

## Abbreviations

BDI-II: Beck’s Depression Inventory II
EQ-5D-5 L: Instrument to assess health-related quality of life
FES-I: Falls Efficacy Scale-International
MDS-UPDRS: Movement Disorders Society-sponsored Unified Parkinson’s Rating Scale
MMSE: Minimal Mental State Examination
PD: Parkinson’s disease
TMT: Trail Making Test
TUG: Timed-Up-and-Go test
